# Supplementary material for: O-GlcNAcylation mediates Wnt-stimulated bone formation by rewiring aerobic glycolysis
Source: EMBO Rep. 2024 Sep 10;25(10):4465–87. doi: 10.1038/s44319-024-00237-z (PMC11467389; doi:10.1038/s44319-024-00237-z)
Supplement: Supplementary file 1 — Appendix [file 44319_2024_237_MOESM1_ESM.pdf]

Appendix for: O-GlcNAcylation mediates Wnt-stimulated bone formation by rewiring aerobic glycolysis

Table of Contents

Appendix Table S1. Related to Fig. 3. 2

Appendix Table S2. Related to Fig. 3. 2

Appendix Table S3 3

Appendix Table S4 4

Appendix Table S1. Related to Fig. 3.

μCT analyses of distal metaphysis of the femurs

Group	BV/TV (%)	Tb.N (1/mm)	Tb.Th (mm)	Tb.Sp (mm)
Ctrl+PBS	22.50±5.34	5.48±0.21	0.06±0.008	0.18±0.009
OgtCKO+PBS	10.14±2.11*	3.93±0.71*	0.044±0.004	0.26±0.059*
Ctrl+Scl-Ab	43.35±6.52**	6.67±0.36**	0.08±0.009**	0.13±0.013
OgtCKO+Scl-Ab	26.62±1.82***	5.54±0.30***	0.066±0.007***	0.17±0.011

Data are shown as mean ± SD, n=5.

BV/TV = bone volume over tissue volume; Tb.N = trabecular number; Tb.h = trabecular thickness; Tb.Sp = trabecular space. Data acquired from 100 μCT slices (1.31 mm) immediately below the distal femoral growth plate. *p<0.05, OgtCKO+PBS versus Ctrl+PBS; **p<0.05, Ctrl+Scl-Ab versus Ctrl+PBS; ***p<0.05, OgtCKO+Scl-Ab versus Ctrl+Scl-Ab, two-way ANOVA followed by Tukey's multiple comparisons test.

Appendix Table S2. Related to Fig. 3.

μCT analyses of diaphyseal cortical bone of the femurs

Group	Ct.Th (mm)	Tt.Ar (mm2)	Ct.Ar (mm2)	Ct.Ar/Tt.Ar (%)
Ctrl+PBS	0.15±0.014	1.60±0.19	0.62±0.087	38.39±1.63
OgtCKO+PBS	0.11±0.016*	1.31±0.099*	0.41±0.062*	31.12±3.23*
Ctrl+Scl-Ab	0.21±0.008**	1.92±0.20**	0.90±0.10**	46.93±0.94**
OgtCKO+Scl-Ab	0.11±0.011***	1.43±0.13***	0.45±0.055***	31.30±2.01***

Data are shown as mean ± SD, n=5. Ct.Th = cortical thickness; Tt.Ar = total area; Ct.Ar = cortical area; Ct.Ar/Tt.Ar = cortical area over total area. Data acquired from 50 μCT slices (0.655 mm) of the mid-diaphyseal of the femur. *p<0.05, OgtCKO+PBS versus Ctrl+PBS; **p<0.05, Ctrl+Scl-Ab versus Ctrl+PBS; ***p<0.05, OgtCKO+Scl-Ab versus Ctrl+Scl-Ab, two-way ANOVA followed by Tukey's multiple comparisons test.

Appendix Table S3

siRNA sequences

Gene name		sequence（5' to 3'）
si*NC*	sense	UUCUCCGAACGUGUCACGUTT
si*NC*	anti-sense	ACGUGACACGUUCGGAGAATT
si*Gfat1*	sense	GGAAUCUUGCCAAAUCUGUTT
si*Gfat1*	anti-sense	ACAGAUUUGGCAAGAUUCCTT
si*Ogt*	sense	GCUGAUGUCUGCCUGGAUAdTdT
si*Ogt*	anti-sense	UAUCCAGGCAGACAUCAGCdTdT
si*Oga*	sense	GCUGCACGAGAAUAUGAAAdTdT
si*Oga*	anti-sense	UUUCAUAUUCUCGUGCAGCdTdT
si-*β-catenin*#1	sense	CAGUUGUCAAUUUGAUUAATT
si-*β-catenin*#1	anti-sense	UUAAUCAAAUUGACAACUGTT
si-*β-catenin*#3	sense	GGGCAGUAUGCAAUGACUATT
si-*β-catenin*#3	anti-sense	UAGUCAUUGCAUACUGCCCTT
si*HK2*	sense	GGACAGAACAUGGCGAGUUTT
si*HK2*	anti-sense	AACUCGCCAUGUUCUGUCCTT

Appendix Table S4

Primer sequences.

Gene name		sequence（5’ to 3’）
β-actin	Forward	AGATGTGGATCAGCAAGCAG
	Reverse	GCGCAAGTTAGGTTTTGTCA
Alpl	Forward	CCAACTCTTTTGTGCCAGAGA
	Reverse	GGCTACATTGGTGTTGAGCTTTT
Bglap	Forward	CCAACCGAGTCATTTAAGGCT
	Reverse	GCTCACGTCGCTCATCTTG
Ogt	Forward	TCCTGATTTGTACTGTGTTCGC
	Reverse	AAGCTACTGCAAAGTTCGGTT
Gfat1	Forward	TGGTGTGCGGAGTGAACATAA
	Reverse	GTGTGCTCTATCACGGCACTT
Hk2	Forward	CTAAGGGGTTCAAGTCCAGTGG
	Reverse	AGACCAATCTCGCAGTTCTGA
Runx2	Forward	CCAACCGAGTCATTTAAGGCT
	Reverse	GCTCACGTCGCTCATCTTG
Sp7	Forward	CCCTTCTCAAGCACCAATGG
	Reverse	AAGGGTGGGTAGTCATTTGCATA
Ibsp	Forward	CAGAGGAGGCAAGCGTCACT
	Reverse	GCTGTCTGGGTGCCAACACT
