# Supplementary material for: O-GlcNAcylation mediates Wnt-stimulated bone formation by rewiring aerobic glycolysis
Source: EMBO Rep. 2024 Sep 10;25(10):4465–87. doi: 10.1038/s44319-024-00237-z (PMC11467389; doi:10.1038/s44319-024-00237-z)
Supplement: Supplementary file 11 — Expanded View Figures [file 44319_2024_237_MOESM11_ESM.pdf]

Expanded View Figures

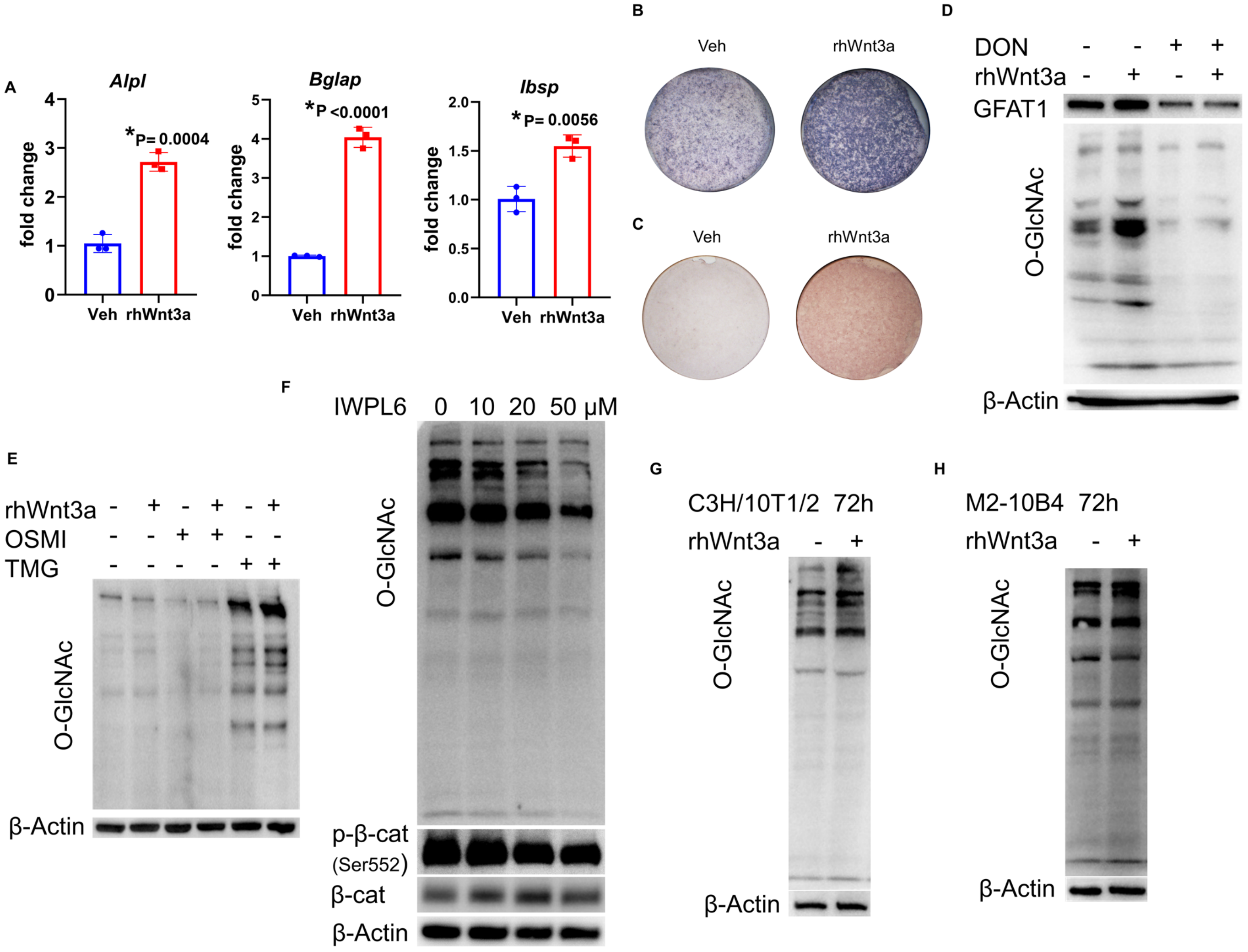

Figure EV1. Wnt3a increases protein O-GlcNAcylation that mediates Wnt-induces osteogenesis in vitro.

(related to Figs. 1 and 2) (**A**) ST2 cells were treated with 50 ng/ml rhWnt3a for 3 days and relative expression of osteogenic genes was tested by qPCR. Each dot represented one single experiment. Error bars: mean ± SD. *$p < 0.05$; $n = 3$, biological replicates (two-tailed Student's t-test). (**B**) ALP staining, and (**C**) Alizarin red S staining were performed after rhWnt3a treatment for 3 days and 14 days, respectively. (**D**) ST2 were pretreated with GFAT antagonist DON for 6 h and then administrated with rhWnt3a for 2 days. GFAT1 and O-GlcNAc levels were detected by Western blotting. (**E**) ST2 were pretreated with OGT and OGA inhibitors, OSMI and TMG, respectively for 6 h and then administrated with rhWnt3a for 2 days. O-GlcNAc levels are detected by Western blotting. (**F**) ST2 cells were pretreated with IWPL6 for 2 days, and O-GlcNAc, phospho-β-catenin(Ser^{552}) and total β-catenin levels were detected by Western blotting. (**G**, **H**) O-GlcNAcylation expression levels of total proteins in C3H/10T1/2 and M2-10B4 cells after 72 h induction by rhWnt3a.

© The Author(s)

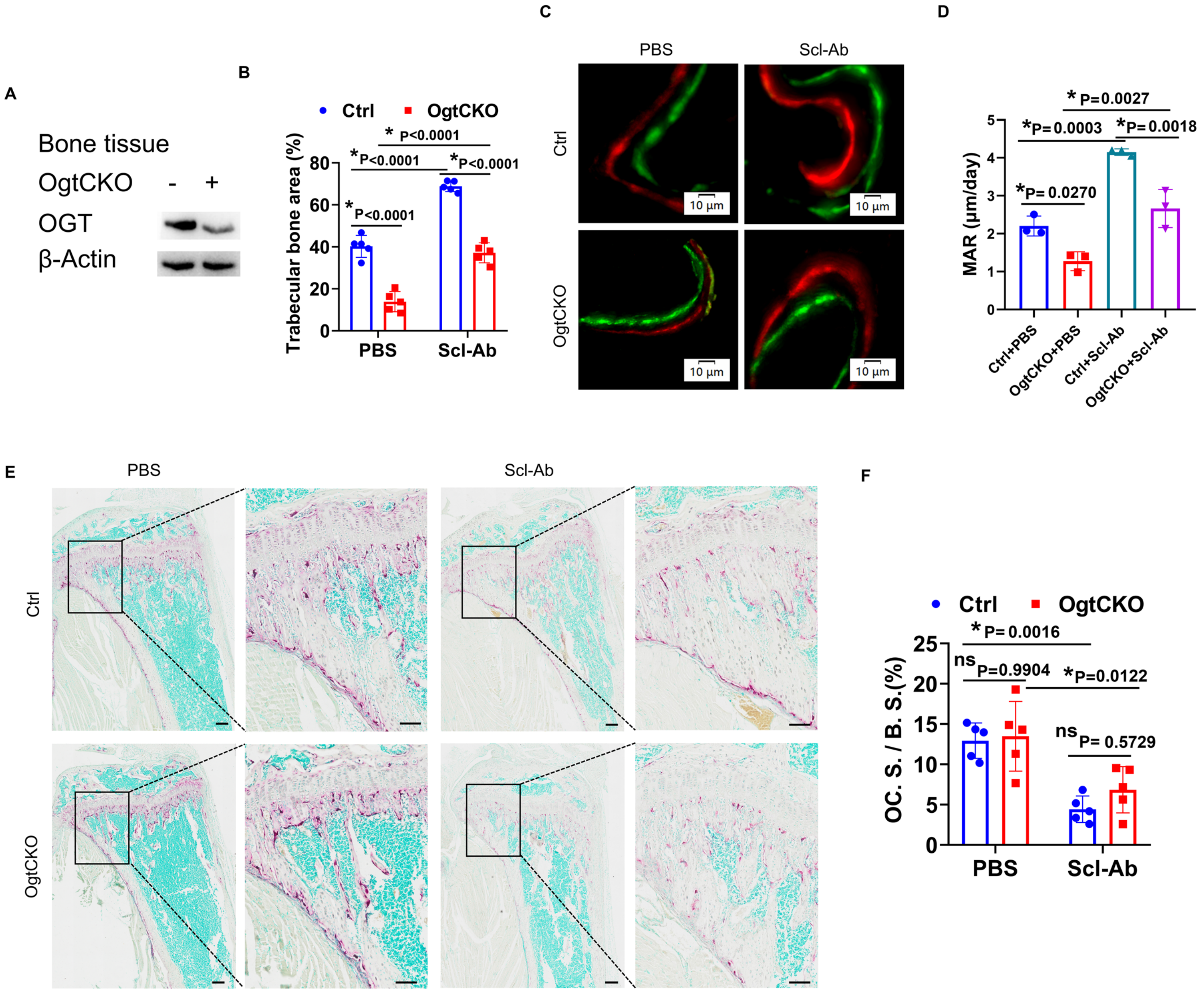

Figure EV2. Deletion of O-GlcNAcylation in osteoblast-lineage cells diminishes Wnt-induced bone formation.

(related to Fig. 3) (**A**) An examination of OGT protein was conducted in femoral and tibial diaphysis tissues from Ctrl mice and OgtCKO mice. (**B**) Quantification of proximal tibial trabeculae in Fig. 3H. (**C**) Representative images of calcein–alizarin red double labeling in the trabecular bone of femurs. Scale bar, 10 μm. (**D**) The quantification of the Mineral Apposition Rate (MAR) of trabecular bone is shown. (**E**) TRAP staining for the tibia from Ctrl or OgtCKO mice with or without Scl-Ab injection, scale bar, 200 μm. Boxed area is shown at a high magnification to the right, scale bar, 100 μm. (**F**) Osteoclast (TRAP-positive multinucleated cells) surface normalized to bone surface (OC. S./B. S.) is shown. The ROI was around 0.09 mm^2 region (300 × 300 μm) underneath the growth plate. Each dot represented one animal. Error bars: mean ± SD. *$p < 0.05$, ANOVA followed by Tukey's multiple comparisons test.

 © The Author(s)

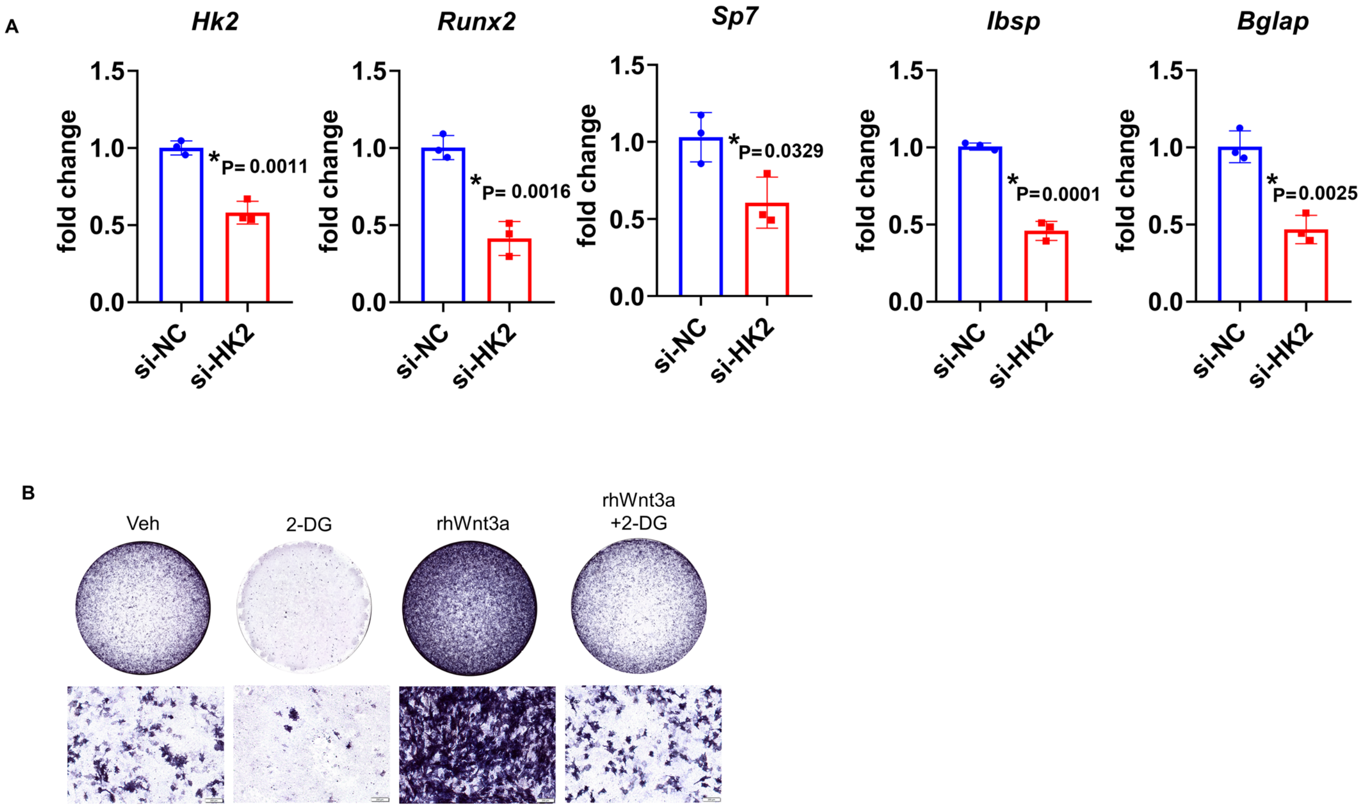

Figure EV3. O-GlcNAcylation is indispensable for Wnt3a-increased aerobic glycolysis.

(related to Figs. 5 and 8) (**A**) Osteoblastic gene detection by qPCR in the absence of Hk2. Each dot represented one single experiment. Error bars: mean ± SD. $*p < 0.05$; $n = 3$ (two-tailed Student's t-test). (**B**) ST2 cells were pretreated with 2-DG for 1 day and then exposed to rhWnt3a for 3 additional days. Then ALP staining was performed.

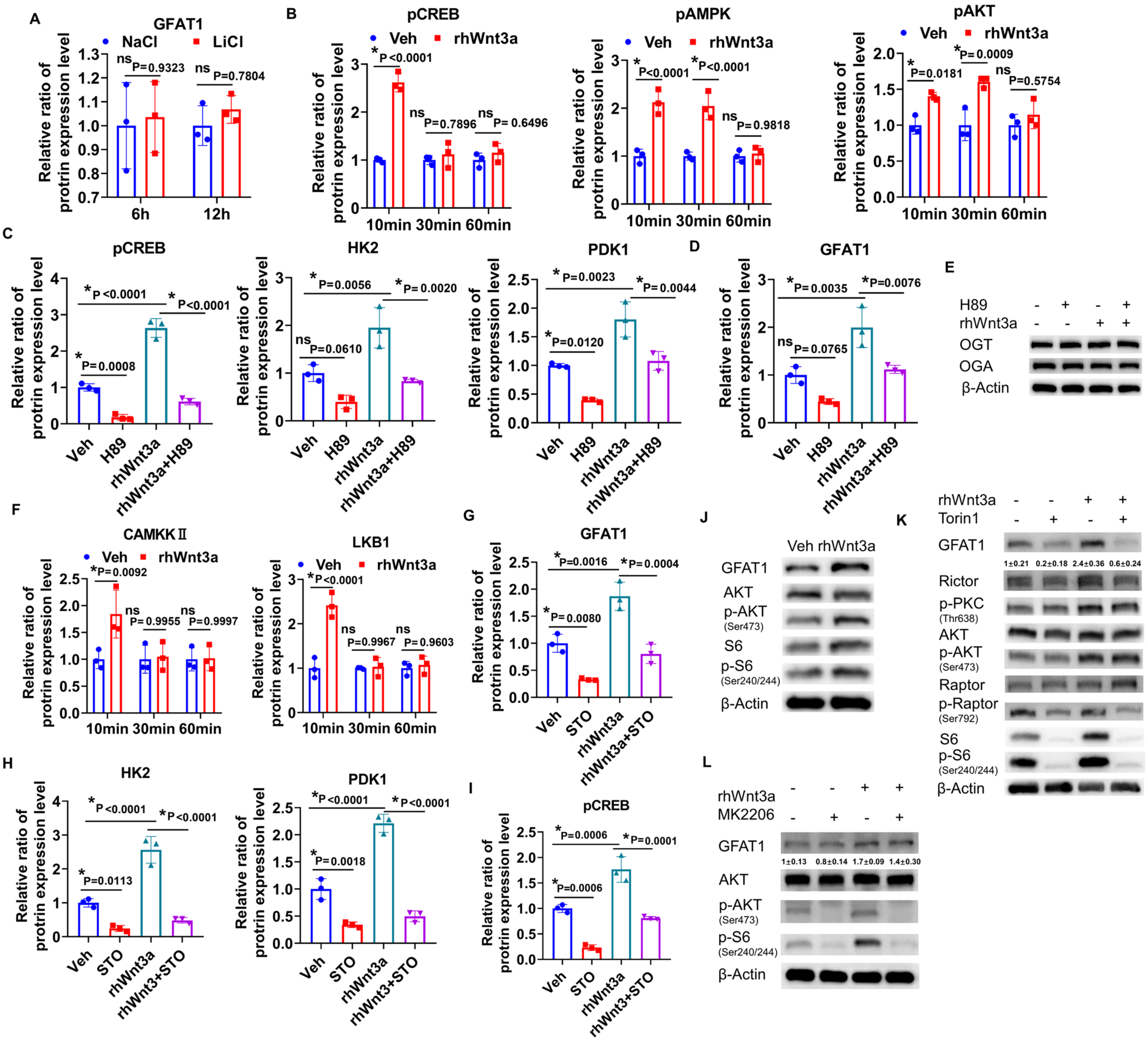

Figure EV4. Wnt rapidly increases O-GlcNAcylation via the Ca^{2+}-PKA-GFAT1 axis.

(related to Fig. 7) (**A**) Quantification of GFAT1 protein shown in Fig. 7A. (**B**) Quantification of pCREB, pAMPK, and pAKT proteins shown in Fig. 7B. (**C**) Quantification of pCREB, HK2, and PDK1 proteins shown in Fig. 7C. (**D**) Quantification of GFAT1 protein shown in Fig. 7D. (**E**) ST2 cells were treated with rhWnt3a, H89, or both for 6 h. OGT and OGA expression levels were detected. (**F**) Quantification of CAMKKII and LKB1 proteins shown in Fig. 7F. (**G**) Quantification of GFAT1 protein shown in Fig. 7G. (**H**) Quantification of HK2 and PDK1 proteins shown in Fig. 7H. (**I**) Quantification of pCREB protein shown in Fig. 7K. (**J**) The mTOR downstream targets expression in response to rhWnt3a. (**K**) The mTOR inhibition decreases GFAT1 protein level. (**L**) The GFAT1 expression detection after the mTORC2 inhibition. All data are shown as mean ± SD, $n = 3$, biological replicates. *$p < 0.05$, (**A**, **B**, **F**) two-way ANOVA followed by Sidak's multiple comparisons test, (**C**, **D**, **G**, **H** and **I**) one-way ANOVA followed by Tukey's multiple comparisons test.

 © The Author(s)

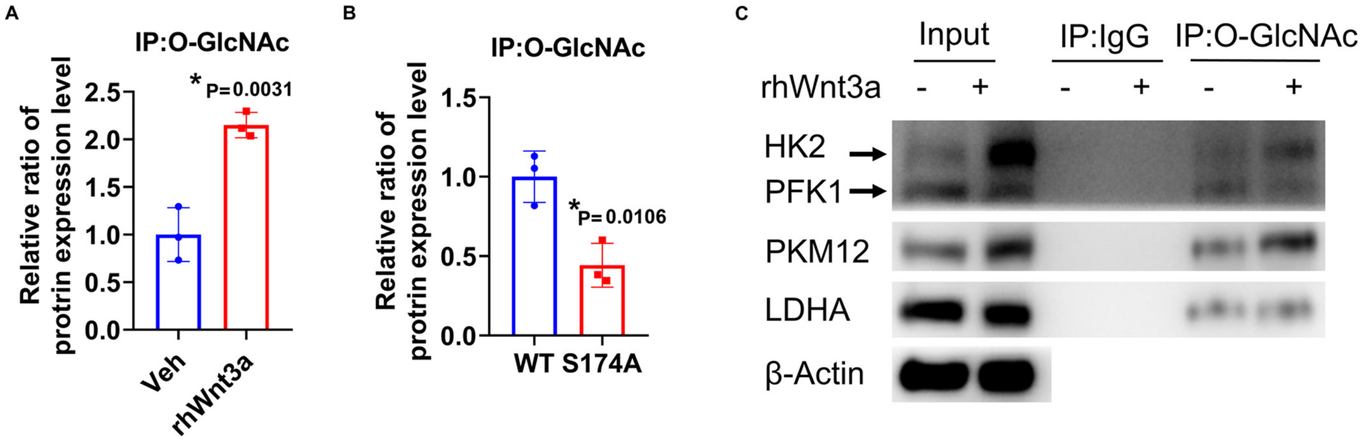

Figure EV5. O-GlcNAcylation on the Ser[174] site stabilizes PDK1 and facilitates Wnt3a-induced osteogenesis.

(related to Fig. 8) (**A**) Quantification of the IPed O-GlcNAc protein in Fig. 8B. (**B**) Quantification of the IPed O-GlcNAc protein in Fig. 8E. (**C**) Immunoprecipitation with endogenous O-GlcNAc and detection with HK2, PFK1, PKM12, and LDHA, respectively. Error bars: mean ± SD. *$p < 0.05$; $n = 3$, biological replicates (two-tailed Student's t-test).
